# Book review: Epigenetics (second edition, eds. Allis, Caparros, Jenuwein, Reinberg)

**DOI:** 10.3389/fgene.2015.00315

**Published:** 2015-10-26

**Authors:** Krassimir Yankulov

**Affiliations:** Department of Molecular and Cellular Biology, University of GuelphGuelph, ON, Canada

**Keywords:** epigenetics, education, model organisms, epigenome, chromatin, molecular biology

Epigenetics is a dynamic and well-established branch of genetics. It deals with heritable traits, which are not transmitted by the sequence of DNA but rather by the state of chromatin. The evolving landscape of epigenetic research has been reviewed in many excellent books and monographs. Amongst these, the second edition of *Epigenetics* by Cold Spring Harbor Laboratory Press (Allis et al., 2015) stands out as one of the most comprehensive references for all major developments and perspectives in the field. Built upon the foundation of the first edition (published in 2007), this new edition continues to deliver a solid basic knowledge of various epigenetic processes in model organisms (including yeasts, ciliates, plants, insects, and mammals), of gene imprinting, of dosage compensation, of DNA methylation, and histone modifications. Twelve new chapters track the recent developments in epigenetic processes in cancer, neuronal development, and mental illness, in responses to the environment and in long-range chromatin interactions. All chapters are written by prestigious researchers and are nicely organized to start with a brisk summary followed by a short overview and heavily and richly illustrated main text. In this respect, the book targets a broad audience and can easily serve as an educational resource in specialized higher level undergraduate and graduate university courses or as a reference for advanced level scientists. Its breadth of topics makes it a compulsory item on the shelf of every lab that is closely or remotely involved in epigenetic studies.

The first chapter (written by G. Felsenfeld) gives us a brief history of epigenetics. This history is neither short nor simple. It began some 60 years ago with the notion that all cells in a multicellular organism contain the same DNA, but the gene expression patterns in differentiated tissues dramatically vary. In chapter 3 the editors take this lead and provide an extended and comprehensive overview of chromatin structure and how its transmission, remodeling, and modifications regulate distinct gene expression programs from the same genomic DNA. The editors place a special emphasis on “genetics vs. epigenetics.” They comment on the “missing heredity” in certain enigmatic phenomena and human pathologies and introduce the idea that all these are caused by epigenetic mechanisms. This very entertaining chapter could easily be published as a stand-alone guide to the universe of epigenetic heredity. At the same time, it is a long summary of the more detailed material that is extensively discussed in the remaining 33 chapters of the book. These chapters occupy more than 800 pages and cover practically every aspect of contemporary epigenetics. They are comprehensive and informative, but at times this massive volume could be overwhelming. Some of the chapters focus on the studies of epigenetic processes in model organisms and what we learn from them. Besides being very enlightening, these chapters are an excellent reference for the names and the functions of the multiple protein complexes, enzymes, gene names and homologs in different organisms. Every researcher in the field will appreciate the thoroughness of this compiled and updated information. Other chapters encompass general phenomena observed in many species, such as the effects of the environment on the epigenome or the roles of non-coding RNAs in the regulation of chromatin structure. Finally, epigenetic aspects of mammalian stem cell biology, development, immunity, genetic disease, and cancer are discussed. These particular chapters are perfectly suited for an aspiring medical student or for graduate students in the broader fields of developmental biology, immunology, or cancer biology. A missing theme in this textbook is a dedicated chapter(s) on the emerging trends in pharmacology to target and modulate the activity of enzymes that are involved in various epigenetic processes.

A most interesting addition to the second edition of *Epigenetics* are the short 2–3 page articles written by junior scientists who have already left their mark in the field. They contain only one figure and emphasize a single message. This simplicity makes them an excellent additional reading material for undergraduate courses. These short essays are compiled in chapter 2 and give a historic twist and sometimes a personal perspective to recent ground-breaking discoveries. The topics span from short and long non-coding RNAs to histone modifications, from histone modifying enzymes to chromosome folding and cellular reprogramming.

At the end of the book we find two very useful appendices. The first one lists several well-maintained web resources and databases that will continue to update us with information after the publication of this edition of Epigenetics. The second appendix leads us through the maze of documented histone modifications and their known functions.

A true value of this book is its reader-friendly approach to the presentation of the material. For example, it is not easy to understand how non-coding RNAs act in the regulation of chromatin structure or how cell type specifications are determined by histone modifications. However, the editors and authors have made everything possible to unburden the reader. The text systematically and meticulously pays attention to the timing and the functional significance of each specific histone modification, to the function of each enzyme involved in this modification or to the interactions between short RNAs and chromatin remodeling factors, and so on. All these messages are accompanied by excellent illustrations. Thus, even an uneducated reader can understand very complex processes and easily pinpoint the activity of each factor in the process. For this reason I recommend this book as a reliable teaching material for all high level courses on genetics or epigenetics.

In summary, the second edition of *Epigenetics* by CSHL Press has met and exceeded the expectations for a reference book or a textbook. Apart from its intimidating volume and breadth, it has no apparent flaws and is likely to be the key book in the field for years to come. It is comprehensive and is loaded with useful details and substantial information. It targets a very broad audience including undergraduate and graduate students, university professors and researchers in the field of genetics, chromatin structure, or epigenetics.

## Funding

Research in Yankulov lab is supported by Natural Sciences and Engineering Research Council of Canada, grant # PGPIN-2015-06727

### Conflict of interest statement

The author declares that the research was conducted in the absence of any commercial or financial relationships that could be construed as a potential conflict of interest.
